# A need for systems thinking and the appliance of (complexity) science in healthcare

**DOI:** 10.1016/j.fhj.2024.100185

**Published:** 2024-09-13

**Authors:** Mark Wright

**Affiliations:** University Hospitals Southampton, United Kingdom

**Keywords:** Flow, Complexity, Systems thinking, Game theory

## Abstract

Hospitals represent complex adaptive systems where interactions and relationships of different components both affect and shape the way they work simultaneously. Pressures on hospitals determine how they behave and many of the problems seen in the NHS and indeed other health services can be viewed through the lens of complexity science and systems thinking. ‘Flow’ of patients through the hospital can be seen as an indicator of how well the hospital ‘system’ is working. The better flow is, the more patients can be treated and the less time is spent waiting in the various queues that accrue around the hospital,

In this article, we explore the impact of these disciplines on patient flow and examine how short-term and overly simple solutions can exacerbate problems in the health service, despite the best intentions of those working in it. Many of today’s problems can be described in terms of ‘system archetypes’ and ‘game theory’. Understanding this may lead to improvement in how services are redesigned to solve these problems.

‘Bring back the matrons’, ‘get rid of the managers – let the doctors and nurses run the place’. Armchair experts are everywhere. The problem is when politicians and people in leadership positions start acting on their advice.

Healthcare demand and waiting times are rising. More people attend the emergency department (ED). At the same time, NHS backlogs demand greater elective activity. There is an urgent pressure to ‘do something’ to fix matters, but it is not simple to easily introduce change based on perceived cause and effect. The reasons for this are discussed in this paper.

Healthcare is complex. Hospitals are complex adaptive systems (CAS) that simultaneously shape, and are shaped by, multiple interacting components and relationships. Complexity science, and its systems thinking component, examines systems or models whose simple parts interact in multiple ways and demonstrates how simple, local rules then lead to the emergence of a global behaviour which is non-linear and not predictable from the actions of those parts.^1^ CAS behave as they do because they have evolved to in relation to yesterdays pressures/problems and their solutions.

Patient ‘flow’ is an example of this complexity. If flow is poor, the hospital grinds to a halt, people queue in ED, we can’t admit elective patients, people are unhappy and, most importantly, there is risk to patients.^2^ Flow is not constant for all conditions of hospital and can speed up or slow down. As the hospital becomes fuller and patients become more scattered, with greater numbers per team, flow slows down^3^ as those teams become less efficient, eventually leading to the system becoming overwhelmed – a kind of ‘Tetris effect’ .^4^

## Complexity

Many analogies can be used to model flow, including comparisons with motorway traffic, fluid in a pipe, economics and even particle physics, among a host of others. Within complexity science are mathematics that can model these phenomena.^1^ However, the models are universally inadequate because the equations turn out to be non-linear with so called ‘sensitive dependence on initial conditions’, meaning that any small difference leads to unpredictable ‘chaotic’ outcomes.^5^ This has been well demonstrated in healthcare, looking at prediction of length of stay (LOS) .^6^ Correctly assigning patients at a high risk of long LOS to the correct discharge pathway is very difficult.

**Figure:**
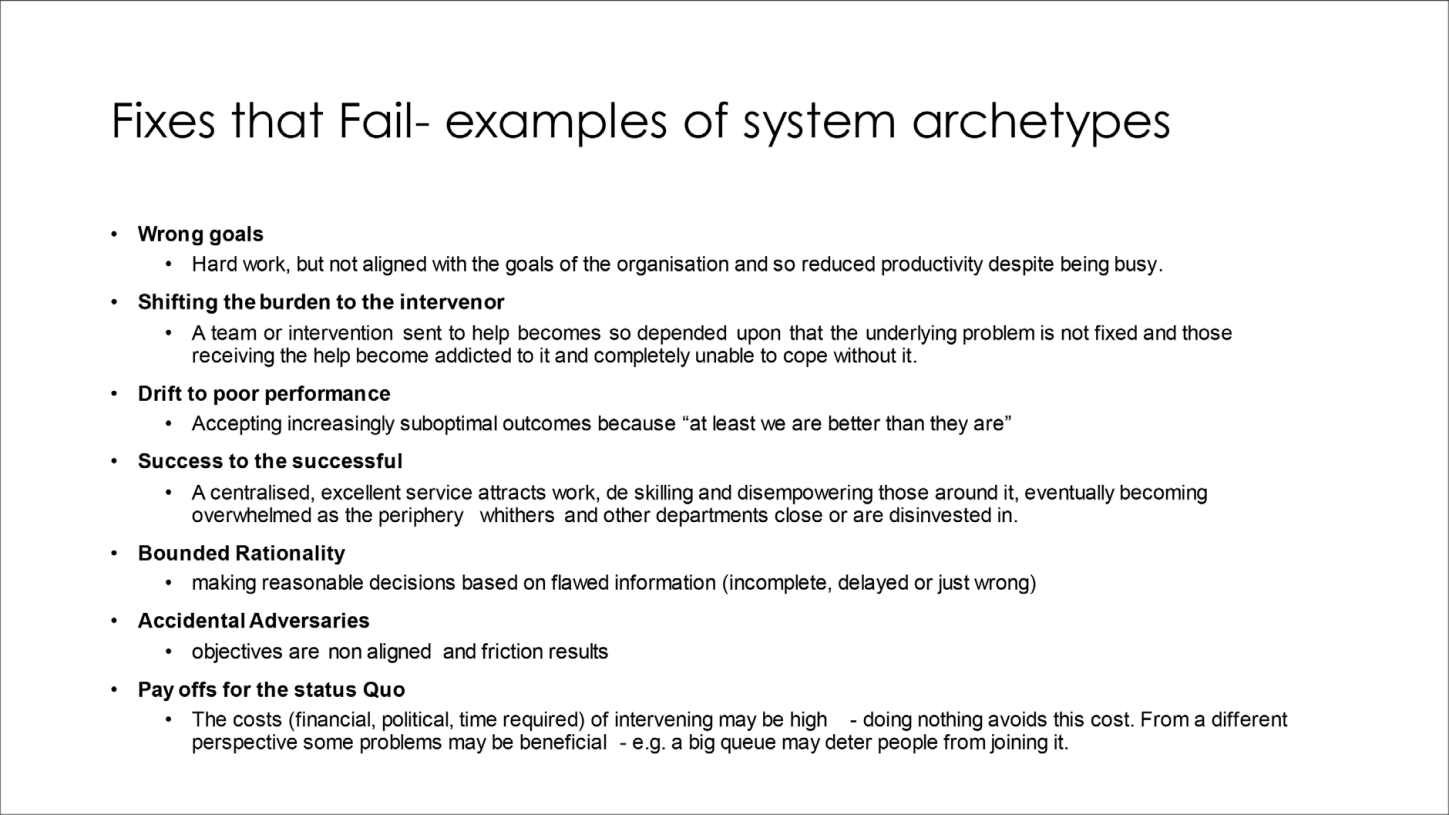

**Glossary of terms**

• Non-linearity
  ∘ Change of output is not proportional to the change of input (and not predictable)
• Emergence
  ∘ When a complex entity has properties or behaviours that its parts do not have on their own, order emerges at the ‘edge of chaos’, co-evolving with external pressures from bordering systems.
• Complex adaptive systems
  ∘ A network of simple interacting components whose combined overall behaviour is not predictable from the individual parts. This gives rise to emergence of new behaviours in the face of selection pressure.
• Sensitive dependence on initial conditions
  ∘ In a non-linear system, minor changes in the starting conditions give rise to very different and often unpredictable outcomes.
• The ‘tragedy of the commons’
  ∘ Multiple actors slightly overexploit a shared resource for their own benefit, unaware that others are doing the same until that resource collapses.
• The ‘prisoner’s dilemma’
  ∘ Classic game theory model where two players can ‘cooperate’ with or ‘betray’ each other without knowing what the other will do. Best outcome is for both to ‘cooperate’, but for each player there is a payoff for ‘betrayal’ in case they themselves are ‘betrayed’.

Alt-text: Unlabelled box

Think about flow like the blood. It is affected by three main factors – 1. The blood itself (patients) has a distribution of viscosity (complexity and comorbidity), reflecting the likelihood of getting stuck. 2. Pressure at each end of the vessel (in vs out) represents political, community and public factors with their own variability. 3. The vessel walls (pathway design and the human operators). Pinch points, policies and behaviours can speed or slow flow and represent friction. Multiple interconnected parallel pathways add further complexity. When smooth flow deteriorates into turbulence (very difficult to describe with maths), drag increases, leading to clots in the system.

As clinicians or managers in hospitals we have little control over the external factors contributing to the type of patients admitted. The best we can do to influence patient flow is to control our internal environment.

Many policies, eg standard pathways, timely discharge paperwork, discharge lounges, criteria-led discharge, electronic information sharing, home before lunch initiatives, discharge ward rounds at weekends, and many more, should facilitate flow, but across the whole system, fail to show impact and consequently productivity doesn’t increase.

## Systems thinking – describing the fixes that fail

Movement of patients through the hospital and timely discharges don’'t seem to work as they should. One reason for this is human behaviour and this can be examined through the lens of system thinking and system archetypes or traps-*‘fixes that fail’.**^7^* This leads to behaviours which will be recognisable where you work.

Examples of this are IT ‘patches’, which deliver ‘safety’ by increasing accountability but inadvertently increase clinician workload and decrease job satisfaction – an example of the ‘wrong goals archetype’. Patches get laid on top of one another because the things they aim to fix are seen in isolation of the whole system.

Zero tolerance safety culture can lead to excessive checks which have a reducing rate of return in terms of impact, slowing the system and provide risk to patients not yet able to get into the system. Fear of punishment when things go wrong drives decision paralysis because of risk aversity. Measures to remove all risk, no matter how small, lead to problems accruing elsewhere in the system – increasing delays and ultimately depriving those in the elective or ED queue (who we can’t see) of healthcare, paradoxically exposing them to risk.

Often the fix fails because of ‘policy resistance’ from the human agents responsible for delivery. These human agents are not out to derail the health service, but are instead acting with the best of intentions. Their actions, however, show ‘bounded rationality’ – making reasonable decisions based on flawed information (incomplete, delayed or just wrong). For example, the nurse in charge of the ward can’t see the queue in ED, just their own busy ward and so may delay declaring an empty bed. They make choices that they can live with for now based on their pre-existing biases. This leads to a ‘tragedy of the commons’ .^8^

Examples in hospital are numerous:

- Not doing discharge papers in a timely way – seen as an unimportant clerical task by doctors, therefore getting left until the end of the day. This is also an example of the ‘wrong goals’ and ‘accidental adversaries’ archetypes; the trust’s objectives are non-aligned with those of the doctors and friction results. Extra information is loaded onto the discharge summary as a means of data collection, lengthening the process and reducing the appeal of doing them, delaying discharge.
- Not discharging people ‘because you just end up getting more’ is a frequently heard sentiment with ward based teams.
- Keeping people a few extra days to ‘keep them safe’. (availability bias- can only see the patient in front of them- not those waiting to come in who are not ‘safe’)
- Holding onto ‘well’ patients instead of sending them to discharge lounges because releasing them will bring sicker patients from ED.

These actions, although individually amounting to just a few patients, when multiplied across the trust lead to a big reduction in flow and a big rise in bed occupancy and LOS.

## Game theory as applied to the NHS

These behaviours can also be seen through the lens of game theory^9^ with similarities to the prisoner’s dilemma. A ward will be ‘punished’ with lots of patients from ED if they are ‘honest’ about empty beds but no one else is, because they will be seen as the only destination. However, if everyone was honest at the same time, the ED patients could be shared out and sent to the appropriate ward.

*Youssarian: ‘From now on I'm looking after number 1.’*
*Psychiatrist: ‘What if everyone thought like that?’*
*Youssarian: ‘Then I'd be a damned fool to think otherwise’*
*Catch 22*^10^

Within the ‘system’, localised responses to problems and attempts to align with hospital priorities lead to various system archetypes. As medicine and nursing have become more specialised, new system traps have emerged. *‘shifting the burden to the intervenor*’ whereby a specialist team (eg safeguarding, diabetes outreach, acute pain, tissue viability or even just different medical specialties) becomes so key to managing a problem that generalists become addicted to their service and lose confidence with even simple things outside their core expertise. This leads to delays as the now-overwhelmed specialists take too long to come and see the patients, creating pinch points.

There are many silos in hospitals and because everyone is working hard, they can’t see what others are doing. Multiple actors pull in different directions. Whenever there is a boundary, complexity increases as responsibilities are unclear and process leads to delay with the possibility of chaos at these borders. For example, we know that discharges at the weekends are lower than in the week and this is not rocket science to explain – the hospital is not functioning the same. Specialty teams lead to pools of patients not fitting various model of care – delays occur negotiating over who takes them, followed by further delays collecting specialist team opinions which may involve ‘pontificatory care’ style advice with long lists of tests to be done before they come. We all need to have skin in the game.

‘Payoffs for the status quo’. A permanent state of crisis perpetuates the need to be in crisis mode –giving power to different groups. Leaders should question their motives and those of politicians. Avoiding investment needed for change is a powerful reason to do nothing, backed up by ever-pervasive financial crises. Big queues deter people from joining them.

## The solutions to these tragedies of the commons?

People need to be aware of their behaviours and these traps so the better to avoid them. Root causes of poor flow need to be identified and changes made on a balance of best overall outcome. We should do the more difficult thing of carefully redesigning things for sustainability – fix the underlying issue – simplify the discharge documents, reduce the ease of access to specialist teams for simple things and (re)educate the work force.

Challenge ‘helicopter parenting’ by managers and hierarchy, as these cause pinch points. Trust professionals, empowering the various subsystems while ensuring that they share the overall system goal. Empowerment comes by acknowledging that all care is a ‘model’ and agreeing how to deal with events that occur as a result of ‘outlying factors’. Poor outcome investigations should start from the premise that a correct decision based on the available (but wrong) information is not a sin.

Blurring boundaries – empowerment to use general medical skills within teams alongside specialist ones – GPs in ED, MOP (medicine for the older person) physicians on orthopaedic and general surgical wards. Senior cover at night and weekends – not popular, but evidenced during the recent strikes. All of this is possible with different use of existing resources.

## Conclusions

In order to change, everyone needs to make explicit choices and be prepared to give things up. Alignment of goals and a clear vision for change articulated to all groups involved, having understood their perpective,^11^ is required. A culture of embedding systems thinking approaches into all service improvements and redesign is needed at all levels of an organisation, with simple core principles aligned with a central goal. Prioritise barriers to discharge, focus on reducing LOS, flow improves, waiting lists fall, adverse incidents related to being in hospital longer than needed fall, safety improves, satisfaction for the patients and outcomes improve.

Thinking about hospital healthcare delivery in a systematic way has huge potential. Complex adaptive systems have multiple balancing feedback loops which have evolved. Often these appear to have little or no role. Slightly too many porters, plentiful admin staff get stripped out for efficiency savings. Then we find that, having ‘got rid of all these managers’ and ‘brought back the matrons’, that doctors and nurses are too busy typing their own letters, pushing beds, navigating an electronic rat maze and trying to sort the mess out themselves that they don’t have time to see patients.

## CRediT authorship contribution statement

**Mark Wright:** Writing – review & editing, Writing – original draft, Conceptualization.

## Declaration of competing interest

The authors declare that they have no known competing financial interests or personal relationships that could have appeared to influence the work reported in this paper.
